# Long-term effects on cardiovascular risk of a structured multidisciplinary lifestyle program in clinical practice

**DOI:** 10.1186/s12872-018-0792-6

**Published:** 2018-04-02

**Authors:** Matthias Lidin, Mai-Lis Hellénius, Monica Rydell-Karlsson, Elin Ekblom-Bak

**Affiliations:** 1 Karolinska Institutet, Department of Medicine, Stockholm, Sweden; 2 Department of Cardiology, Karolinska University Hospital, Stockholm, Sweden; 3 Karolinska Institutet, Department of Clinical Sciences, Danderyd Hospital, Stockholm, Sweden; 4 Ersta Sköndal Bräcke Univerity Collage, Stockholm, Sweden; 5 The Swedish School of Sport and Health Sciences, Stockholm, Sweden

**Keywords:** Lifestyle, Program, Cardiovascular, Risk factor, Multidisciplinary

## Abstract

**Background:**

Cardiovascular disease is still the leading cause of premature death world-wide with factors like abdominal obesity, hypertension and dyslipidemia being central risk factors in the etiology. The aim of the present study was to investigate the effects on cardiovascular risk factors and cardiovascular risk after 6 months and 1 year, in individuals with increased cardiovascular risk enrolled in a lifestyle multidisciplinary program in a clinical setting.

**Method:**

Individuals with increased cardiovascular risk were referred from primary health care and hospitals to a program at an outpatient clinic at a department of cardiology. The program consisted of three individual visits including a health check-up with a physical examination and blood sampling, and a person-centered dialogue for support in behavioural change of unhealthy lifestyle habits (at baseline, 6 months and 1 year). Furthermore, five educational group sessions were given at baseline. Cardiovascular risk was assessed according to Framingham cardiovascular risk predicting model.

**Results:**

One hundred individuals (mean age 59 years, 64% women) enrolled between 2008 and 2014 were included in the study. Waist circumference, systolic and diastolic blood pressure and total cholesterol decreased significantly over 1 year. In parallel, cardiovascular risk according to the cardiovascular risk profile based on Framingham 10-year risk prediction model, decreased with 15%. The risk reduction was seen in both men and women, and in participants with or without previous cardiovascular disease.

**Conclusion:**

Participating in a structured lifestyle program over a year was associated with significant improvement in multiple cardiovascular risk factors and decreased overall cardiovascular risk.

**Trial registration:**

The study is registered at www.clinicaltrials.gov (ClinicalTrial.gov ID: NCT02744157).

## Background

Cardiovascular disease (CVD) is the leading cause of premature death world-wide [1], with abdominal obesity, hypertension and dyslipidaemia being central risk factors in the etiology of CVD. Conversely, modifiable lifestyle habits including physical activity (PA), limited time spent sedentary, a healthy diet and smoking cessation have all been shown to have beneficial effect on CVD risk, mediated partly by improving traditional intermediating CVD risk factors [2–4]. A healthy lifestyle is the first choice for prevention and treatment of CVD [5], and according to the World Health Organization, a healthy lifestyle combined with optimal medical treatment could prevent 75% of all CVD world-wide [6].

Previous Swedish prevention studies have showed that increased PA, decreased sedentary behaviour and a healthier food pattern improved cardiovascular risk factors and decreased CVD risk after 1 months in individuals with high CVD-risk [7, 8]. Multidisciplinary secondary preventive programs with focus on a healthy lifestyle, risk factor management and medication adherence have been proven effective to reduce CVD risk [2, 4, 9, 10]. However, an inadequate risk factor control in patients with coronary disease has been reported, with the large majority of patients not achieving the secondary prevention guideline standards [11]. Furthermore, lifestyle habits have deteriorated over time with an increase in physical inactivity and sedentary time, unhealthy food pattern, central obesity and diabetes, and stagnating rates of persistent smoking [11–16]. Moreover, scientific evaluations of lifestyle interventions in clinical practice are still limited [7, 9], despite the attention given to lifestyle intervention in current CVD guidelines.

In 2008, a one-year structured lifestyle outpatient clinic program at the Karolinska University Hospital setting in Stockholm, with the aim to guide individuals with increased cardiovascular risk to a change in unhealthy lifestyle habits, with a potential subsequent effect on cardiovascular risk. In a previous publication, significant positive changes in multiple lifestyle habits after 1 year was reported, including increased PA and decreased time spent sedentary, a more healthy food pattern and decreased alcohol intake [17].

The aim of the present study is to investigate the effects on cardiovascular risk factors and cardiovascular risk after 6 months and 1 year, in individuals with increased cardiovascular risk enrolled in the above referred structured lifestyle program.

## Methods

### Study population

Individuals with increased CVD risk were referred by their physician from either primary health care or hospital care and enrolled in the structured lifestyle program at Karolinska University hospital between 2008 and 2014. The inclusion criteria were men and women ≥ 18 year presenting at least three of the following risk factors; physical inactivity, unhealthy food habits, present smoking, risk consumption of alcohol, high perceived stress, general overweight, abdominal obesity, dyslipidaemia, high blood pressure, insulin resistance, diabetes or previous CVD. Hence, the program included both primary and secondary prevention and the main focus was to provide support in lifestyle changes in order to reduce cardiovascular risk.

**Fig. 1:**
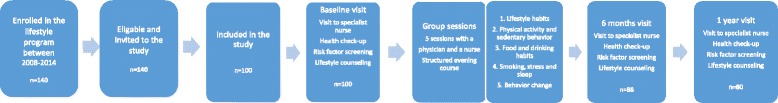
Flowchart with enrolment, inclusion and design of the lifestyle program

The exclusion criteria were inability to understand the Swedish language or to attend the entire program, alcohol addiction, and psychiatric diagnoses due to inability to contribute in group session. All participants provided written consent before inclusion in the study. The study was conducted according to ethical guidelines of the Helsinki declaration and the Good Clinical Practice (GCP) guidelines. The local ethics committee of the Karolinska Institutet approved the study (reference number: 2015/494–31/2). The study was registered at www.clinical-trials.gov (ClinicalTrial.gov ID: NCT02744157). A total of 140 patients were invited to the study, and 100 agreed to participate. A flowchart with enrolment, inclusion and design of the lifestyle program is presented in Fig. 1.

### The structured lifestyle program

The intervention program has previously been described [17]. In brief, the intervention consisted of an individual visit (approximately 1 hour) to a nurse at baseline, after 6 months and 1 year. At the visit, the nurse preformed a health check-up with a person-centered approach using motivational technique for support in behavioural change of unhealthy lifestyle habits. The participant also received a personalized prescription of PA and a pedometer. After the initial visit, participants were offered participation in five educational group sessions led by a nurse and a physician with focus on lifestyle habits; 1) overall lifestyle and health 2) physical activity and sedentary behaviour 3) dietary habits and use of alcohol and tobacco 4) stress and sleeping habits and 5) behavioural change (Fig. 1). Each group session included an initial lecture of the topic followed by open discussion, and consisted of 10 to 15 participants which all were encouraged to bring a friend or relative for support. Participants were also offered a free web-based lifestyle course as a supplement [18].

### Cardiovascular risk factors

At baseline as well as at 6 months, and the one-year visit, height was measured in light-weighing clothes without shoes with a stadiometer to the nearest 1.0 cm, and weight to the nearest 0.1 kg. Body mass index (BMI) was subsequently calculated. Waist circumference was measured to the nearest 0.5 cm in a standing position, midway between the lower rib margin and the iliac crest. Systolic and diastolic blood pressure (BP, in mmHg) was measured in seated position after 10 min rest using standard auscultatory method with a cuff. Resting heart rate was measured manually. A blood sample were drawn from an antecubital vein after overnight fasting, and total s-Cholesterol (mmol/l), s-Low density lipoprotein (LDL, mmol/l), s-High density lipoprotein (HDL, mmol/l) and s-Triglycerides (mmol/l) were assessed by standard methods according to local laboratory routines at Karolinska University Hospital.

### Assessment of general cardiovascular risk profile

Cardiovascular risk was estimated using the general cardiovascular risk profile based on Framingham 10-year risk prediction model [19]. Age, smoking, systolic BP, total cholesterol, HDL-cholesterol and occurrence of diabetes type-2 were entered in the sex-specific multivariable risk factor algorithms and the 10-year probability of developing a CVD was estimated.

### Lifestyle habits and medical records

At all three visits, the participants filled in a questionnaire regarding lifestyle habits and living conditions which has been described in detail previously [17]. In brief, educational level were dichotomized into university degree or not, smoking into daily smoking or not, and risk consumption of alcohol was based on sex-specific evaluations of frequency and quantity of alcohol intake [17]. Time spent sedentary was reported in hours and minutes using the IPAQ short questionnaire [20]. Daily activity was dichotomized into ≥ 30 min per day or less, and exercise habits into ≥ 1 h per week or less. Dietary habits were assessed by fourteen validated questions, covering for example daily intake of vegetables, quality of fat and extra calories from snacks. Stress was dichotomized into getting easily stressed often/almost always or not. CVD and diabetes type 2 diagnoses were derived from the patient medical journal. The participants medication were obtain both from medical records form and by self-reported at the initial visit.

### Statistical analyzes

Data was checked for normality using Shapiro-Wilk test. Normally distributed variables (weight, waist circumference and total cholesterol) are presented as mean ± standard deviation (SD) in Table 2. Repeated measures ANOVA with Greenhouse-Geisser correction and subsequent post-hoc analyses with Bonferroni correction were used to study if the variables differed between the three time points (baseline, 6 months and 1 year). Skewed variables (BMI, systolic and diastolic blood pressure, heart rate, LDL cholesterol, HDL cholesterol, triglycerides and cardiovascular risk according to Framingham CV-risk predicting model) are presented as median with the interquartile range (Q1 to Q3), with Friedman’s 2-way ANOVA by ranks and post-hoc analyses with Bonferroni correction to study if the variables differed between the three time points.

**Fig. 2:**
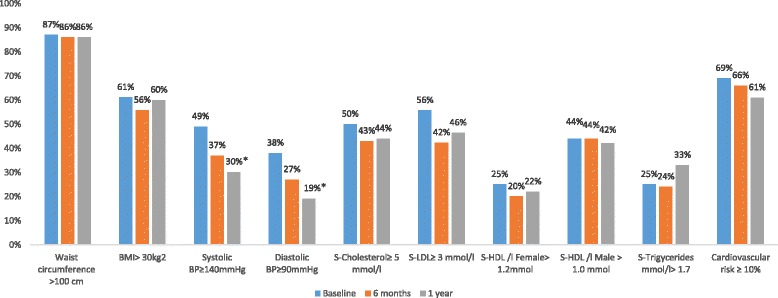
Proportions of participants with risk for each individual CVD risk factors at baseline, after 6 months and 1 year. *Significantly different from baseline ≤ 0.017 with Bonferroni correction for multipel testing

Intention to treat approach was used, and last observation (from baseline or 6 months) was carried forward for missing data for all variables. Further, all CVD risk factor variables were dichotomized according to conventional cut-off points for indication of increased CVD risk (Fig. 2). The prevalence of participants with risk factors values above these cut-offs were compared between baseline, 6 months and 1 year and a 99% confidence interval (CI) was calculated. The 99% CI was used to adjust for multiple testing. Statistical analyses were performed using SPSS (version 24).

## Results

Demographic data at baseline is presented in Table 1. In total, 100 individuals with high cardiovascular risk, mean age 59 years (64% women), were included at baseline. More than one third of the participants had a university degree and living alone was common. The numbers of smokers was low and a minority reported high levels of perceived stress. Almost half of the participants performed less than 30 min of daily activity and spent more than 7 h (480 min) a day sitting. In addition, two-thirds exercised less than 1 hour per week. Eighty-four percent reported low intake of vegetables (a few times per week or less). Thirty-six percent had diagnosed CVD and 21% were diagnosed with type-2 diabetes. Sixty six percent of the participants were on treatment for hypertension and 36% were on statin treatment.

**Table 1:** Demographic data at baseline

	Men	Women	Total
	*n* = 36	*n* = 64	*n* = 100
Age (years)	58.3 ± 9.5	58.7 ± 11.3	58.6 ± 10.6
Social status (single)	11 (31%)	29 (45%)	40 (40%)
No university degree	19 (53%)	31 (48%)	50 (50%)
Daily smoking	4 (11%)	6 (9%)	10 (10%)
Risk consumption of alcohol	3 (8%)	16 (25%)	19 (19%)
Getting easily stress (often/always)	8 (22%)	18 (28%)	26 (26%)
Daily physical activity < 30 min	22 (61%)	24 (38%)	46 (46%)
Exercise < 1 h per week	26 (72%)	41 (64%)	67 (67%)
Sedentary behaviour ≥7 h per day	19 (53%)	28 (44%)	46 (46%)
Low intake of vegetables (less than daily)	34 (94%)	48 (75%)	82 (82%)
History of cardiovascular disease	16 (44%)	20 (31%)	36 (36%)
Diagnosed with Type 2 diabetes	10 (28%)	11 (17%)	21 (21%)
Blood pressure medication	24 (67%)	42 (66%)	66 (66%)
Blood lipid lowering medication	15 (42%)	21 (33%)	36 (36%)

Data is presented as mean for age (SD) and in numbers of individuals (%)

### Changes in cardiovascular risk factors

Waist circumference decreased over 1 year from 108.4 cm at baseline to 105.9 cm at the one-year visit (*p* = < 0.001). Both mean systolic and diastolic BP decreased over 1 year from 135 to 130 mmHg (*p* = < 0.001) and 85 to 80 mmHg (*p* = < 0.001), respectively. Comparing participants with and without BP lowering medication revealed a decreasing trend in systolic and diastolic BP in both subgroups. Total cholesterol decreased from baseline to 6 months (5.1 mmol/l to 4.9 mmol/l, *p* = 0.019), with no further change at the one-year follow-up visit. Similar trends were seen for LDL-cholesterol. No significant trends were seen either for resting heart rate, HDL or triglycerides. The participant’s medication did not change during the intervention (Table 2).

**Table 2:** Cardiovascular risk factors at baseline, 6 months and 1 year

Total parameter	Baseline *n* = 100	6 months *n* = 88	1 year *n* = 80	ANOVA *p*-value
Weight (kg)	93.4 (19.2)	92.5 (19.5)^a^	92.6 (19.8)	0.056
BMI (kg/m^2^)	31.6 (28.3 to 35.5)	31.4 (28.1 to 35.3)^a^	31.1 (28.0 to 34.9)	< 0.001
Waist Circumference (cm)	108.4 (15.0)	106.8 (15.3)^a^	105.9 (15.1)^a, b^	< 0.001
*Men (cm)*	113.5 (14.6)	112.4 (14.3)	111.7 (13.2)^a^	0.099
*Women (cm)*	105.5 (14.6)	103.7^a^(15.1)	102.6^a,b^(15.1)	0.001
Systolic BP (mmHg)	135 (120 to 149)	130 (120 to 140)^a^	130 (120 to 140)^a^	< 0.001
With BP lowering medication* *(n = 66)*	140 (125 to 150)	130 (120 to 140)	130 (120 to 140)^a^	0.002
*No medication (n = 34)*	130 (120 to 140)	120 (119 to 140)	130 (118 to 136)	0.027
Diastolic BP (mmHg)	85 (80 to 90)	80 (75 to 90)^a^	80 (75to 85)^a^	< 0.001
With BP lowering medication* *(n = 66)*	85 (80 to 90)	80 (75 to 90)	80 (79 to 85)^a^	0.006
*No medication (n = 34)*	80 (80 to 90)	80 (80 to 85)	80 (70 to 90)	0.018
Heart rate (bpm)	66 (60 to 76)	68 (62 to 80)	64 (60 to 76)	0.087
Total Cholesterol (mmol/l)	5.1 (1.1)	4.9 (1.1)^a^	4.9 (1.0)	0.019
*With Statins (n = 36)*	4.5 (0.9)	4.3 (0.8)	4.4 (0.9)	0.151
*No statins (n = 64)*	5.4 (1.1)	5.2 (1.1)	5.1 (1.0)	0.081
LDL (mmol/l)	3.1 (2.4 to 3.9)	2.8 (2.3 to 3.9)	2.8 (2.2 to 3.7)	0.065
*With Statins (n = 36)*	2.6 (2.1 to 3.1)	2.4 (2.1 to 2.7)	2.4 (2.0 to 2.7)	0.342
*No statins (n = 64)*	3.6 (2.8 to 4.2)	3.2(2.6 to 4.1)	3.2 (2.6 to 4.0)	0.181
HDL (mmol/l)	1.3 (1.0 to 1.5)	1.2 (1.0 to 1.5)	1.4 (1.0 to 1.6)	0.227
*Men (n = 36)*	1.1 (0.4)	1.1 (0.5)	1.2 (0.6)	0.260
*Women (n = 64)*	1.4 (0.4)	1.4 (0.4)	1.4 (0.4)	0.633
Triglycerides (mmol/l)	1.2 (0.9 to 1.6)	1.3 (0.9 to 1.6)	1.2 (0.8 to 1.8)	0.376

Values are presented as mean (SD) or median (Q1 to Q3)

^a^Significantly different from baseline, *p* < 0.05

^b^Significantly different from 6 months, *p* < 0.05

*BMI* Body Mass Index, *BP* Blood pressure, *LDL* Low density lipoprotein, *HDL* High density lipoprotein

### Changes in cardiovascular risk

Cardiovascular risk decreased significantly from baseline (15.6%) to the one-year follow-up (13.3%) in the total study population, corresponding to a 15% decrease over 1 year (Table 3). Men had a significantly higher estimated risk at baseline compared with women, 23.5% and 10.8% respectively, but the estimated risk decreased significantly in both groups over 1 year (− 22% in men and − 20% in women). Similar results were seen when comparing participants with a history of CVD with those without. In participants with CVD, the mean score decreased with 21% over 1 year, while in participants without CVD, mean score decreased with 28% over the same time period (Table 3).

**Table 3:** Estimated risk of developing CVD according to cardiovascular risk profile based on Framingham 10-year risk prediction model score at baseline, 6 months and 1 year

	Baseline	6 months	1 year	ANOVA *p*-value
Total Framingham risk	15.6 (8.0 to 25.3)	13.7 (6.3 to 21.6)^a^	13.3 (6.3 to 20.8)^a^	< 0.001
Men (*n* = 36)	23.5 (15.6 to 30)	21.6 (15.6 to 29.4)	18.4 (13.3 to 29.4)	0.007
Women (*n* = 64)	10.8 (4.7 to 18.5)	10.0(3.9 to 15.9)^a^	8.6 (4.5 to 15.9)^a^	0.001
CVD (*n* = 36)	23.5 (14.2 to 30)	21.5 (13.7 to 27.5)	18.5 (11.3 to 28.9)	0.033
Without CVD (*n* = 64)	13.5 (5.6 to 18.5)	10 (4.1 to 18.4)^a^	9.7 (4.7 to 15.9)^a^	< 0.001

All values presented as median (Q1 to Q3). ^a^Significantly different from baseline, *p* < 0.05

### Change in individuals cardiovascular risk factors

Changes in proportions (%) of individuals at increased risk for each CVD risk factor is presented in Fig. 2. There was an overall trend towards decreased proportions of individuals at risk for the majority of the CVD risk factors, with a significant lower proportion with high systolic and diastolic blood pressure at 1 year. The proportion of individuals with high levels of total cholesterol, LDL-cholesterol decreased, whereas the proportion of individuals with low HDL-cholesterol levels decreased. The proportions of individuals with high risk according to the cardiovascular risk profile based on Framingham 10-year risk prediction model decreased somewhat, while the proportion of individuals with high waist circumference or BMI were unchanged. The proportion of hypertriglyceridemia increased at the one-year visit.

## Discussion

The main findings in the present study were that participating in a structured lifestyle program over a year was associated with significant improvement in multiple cardiovascular risk factors and decreased overall cardiovascular risk in individuals with increased cardiovascular risk. We noted significantly lower waist circumference, systolic BP, diastolic BP and total cholesterol over 1 year in the participants enrolled in the lifestyle program. Moreover, there was a 15% reduction in estimated probability of developing a CVD within 10 years according the cardiovascular risk based on Framingham risk-score. This reduction was seen in both men and women as well as in participants with or without a history of CVD. In parallel, an overall beneficial change in dichotomized CVD risk factors was observed over year.

There is a call for studies evaluating different approaches for lifestyle interventions in everyday practice with a focus on risk factor management [7, 9, 21]. The present findings are comparable to findings in previous studies. For example, the innovative family-centred preventive cardiology programme MyAction [9] revealed improvements in lifestyle habits as increased PA, improved food habits and improved quality of life and risk factors like blood pressure, lipids and glycemic targets after 1 year in patients with CVD. We previously reported similar results in participants of the present lifestyle program regarding lifestyle habits [17]. While the focus in the MyAction study was on secondary prevention in participants with coronary events, the present study had both a primary and a secondary preventive focus, and included a more heterogeneous study population with different diagnoses and risk profiles. Moreover, Hellénius and co-workers showed in a randomized controlled trial in male participants with high cardiovascular risk, that lifestyle counselling on dietary habits and PA improved several individual risk factors after 6 months, with a reduced estimated cardiovascular risk of 12–14% according to Framingham 10-year risk score [7]. Moreover, 68-year old sedentary individuals with abdominal overweight showed significant improvements in cardiovascular risk factors such as waist circumference, S-cholesterol, ApoB /ApoA1 ratio after 6 months when after participating in a randomized controlled trial to improve PA habits by using physical activity on prescription [8]. In the present study, we also used PA on prescription as one of the tools to motivate the participant to increase the level of daily activity and reduce sedentary time. The OASIS study showed a risk reduction of 47% for developing a new cardiovascular event in individuals with CVD that reporting improvements in lifestyle including more PA and a healthier diet after 6 months [21].

In a previous publication, we aimed at investigating the effect of the intervention program on changes in unhealthy lifestyle habits in the participants of the present study. The results showed that physical inactivity and sedentary time decreased, and diet improved, over 1 year [17]. The decrease in sedentary time and increase in exercise may partly explain some of the favourable changes in risk factors seen in the present study. For example, replacing 2 h of daily sitting with stepping has been associated with a reduction in waist circumference of − 7.5 cm, 11% lower BMI, 11% lower 2-h plasma glucose, 14% lower triglycerides, and 0.10 mmol/L higher HDL cholesterol in overweight adults [22]. Also, a decrease in waist circumference and a more healthy body composition have been shown after increasing daily PA with more exercise and less sitting in an RCT using PAP [8], with similar reduction in systolic and diastolic BP as for the participants in the present study. High systolic and diastolic BP is shown to be reduced by increased exercise levels in previous studies [23, 24], but more recently, also small amounts of low intensity activity have been shown powerful. In a randomized experimental crossover trial in individuals with type-2 diabetes, a first experimental condition of 7 h of uninterrupted sitting was compared to additional experimental conditions in the same participants on two separate days including a) light intensity walking for 3 min every 30 min while seated, and b) simple resistance breaks for 3 min every 30 min while seated [25]. They found a significant reduction both in systolic and diastolic BP after including short breaks with light intensity walk or simple resistance activities compared to continuous sitting. In our study a significant positive-trend in both systolic and diastolic BP were shown both for individuals with hypertensive treatment and not. Also healthy food habits have been shown effective in reducing high BP. For example, the Dietary Approach to Stop Hypertension (DASH) in individuals with hypertension (BP systolic > 160 and diastolic > 90) showed that a more healthy food pattern with more vegetables, fish and vegetable fat lead to reduced systolic and diastolic BP [26].

In the present study, the mean baseline value of S-cholesterol in the total study population was 5.1 mmol/l, which is almost in target according to guidelines for individuals without CVD risk (< 5.0 mmol/L) [5], and was followed by a mean reduction of 0.2 mmol/l (3.9%) after 1 year. This can be compared with previous epidemiological and intervention studies, which has linked a reduction of 1% in total cholesterol to a CVD risk reduction of 2–3% [5, 27]. The reduction was somewhat smaller in participants taking statins (4.5 to 4.4 mmol/l, equal to a 2.2% reduced risk) compared to the participants not taking statins (5.4 to 5.1 mmol/l, equal to a 5.6% reduced risk). The positive effects on cholesterol levels regardless statin treatment on not, indicate that this program could be used in both primary and secondary prevention settings.

There was a significant reduction of 15% between baseline and 1 year follow-up in the overall CVD risk according to general cardiovascular risk profile based on the Framingham 10 year risk estimation. Men and participants with a history of CVD had a significantly higher Framingham risk score at baseline compared to women and non-CVD participants. However, importantly, a significant reduction was seen in both men and women (− 22 and − 20%, respectively), as well as in CVD and non-CVD participants (− 21 and − 28%, respectively) over 1 year. The significant decrease in two of the variables included in the risk estimation, systolic BP and total cholesterol, may explain the large part of the reduction of the risk score at group level. Knowing that these are small groups, the findings could still suggest that the program is suitable for both genders as well as for individuals with a history of CVD and without a history of CVD when targeting overall cardiovascular risk, and could hence be used both in primary and secondary prevention.

In lifestyle interventions like the present one, it is important to have a multidisciplinary approach with different health professionals working together with a focus on behavioural changes of unhealthy lifestyle habits, cardiovascular risk factor management and optimized medication treatment [2, 3, 7, 10]. In the present study, the focus was to strengthen the individual’s ability to change their lifestyle with a person-centered approach, which have shown positive effects on risk factor management and reaching treatment guidelines for individuals with CVD and diabetes [28].

A strength of this study include the evidence based approach of the lifestyle program and the long-term follow-up (1 year). The high attendance to the program (88% at 6 months and 80% at one-year follow-up) indicates that the program is acceptable for individuals with increased cardiovascular risk. Another strength is the beneficial effect on multiple lifestyle style related risk factors, with the subsequent effect on cardiovascular risk over 1 year in both genders and participants with and without a history of CVD. This indicates that the program may be implemented and used in both primary and secondary preventive every day clinical work. There are also some limitations to be mentioned. This study was not a randomized controlled trial, but a prospective observational intervention study without a control arm. The latter hampers the analyses of the causal relationship between program participation and effects on cardiovascular risk, and regression towards the mean affecting the results have to be taken into account. Moreover, individuals who participated in the program may be more motivated, which may limit the generalizability. Another limitation is the small samples size in the subgroup analyzes, potentially influencing the power of the analyses.

We have previously shown positive effects on lifestyle habits and quality of life in individuals with cardiovascular risk both from a primary and secondary approach [17]. The present study indicates potential subsequent effects on cardiovascular risk factors and cardiovascular risk in the same study population. This intervention was initiated and run at an outdoor clinic in a hospital setting with both a primary and secondary preventive focus on cardiovascular risk management, and could serve as one example of how to work with this in clinical practice. Moreover, in our current landscape of finite and limited resources, an economic cost-effectiveness evaluations of the intervention program would be highly valuable for expenditure decisions and enable long-lasting implementation strategies.

## Conclusion

Participating in a structured lifestyle program in a clinical setting led to improvements in multiple individual CVD risk factors and a reduced overall cardiovascular risk according to the cardiovascular risk profile based on Framingham 10-year risk prediction model, in individuals with high cardiovascular risk. The overall CVD risk reduction was seen in both men and women, as well as in both participants with CVD and non-CVD. The high attendance to the program over 1 year together with the results indicates that the program is acceptable for risk factor management in both primary and secondary prevention clinical settings.

## Abbreviations

BMI: Body mass index
BP: Blood pressure
CVD: Cardiovascular disease
HDL: High density lipoprotein
LDL: Low density lipoprotein
PA: Physical activity
PAP: Physical activity on prescription
RCT: Randomised controlled trial
